# Influence of long (16L:8D) and short (8L:16D) photoperiods on blood metabolites and hepatic metabolism in Olive flounder, *Paralichthys olivaceus*

**DOI:** 10.1186/s40064-016-2614-6

**Published:** 2016-06-29

**Authors:** Huafeng Zou, Xianshou Bai, Yuhong Feng, Ying Zhang, Youji Wang, Weiqun Lu

**Affiliations:** Key Laboratory of Exploration and Utilization of Aquatic Genetic Resources, Ministry of Education, Shanghai Ocean University, Shanghai, 201306 China; College of Fisheries and Life Science, Shanghai Ocean University, Shanghai, 201306 China

**Keywords:** Photoperiod, Olive flounder, Hepatic parameters, Energy metabolism, *hsl*, *pepck*

## Abstract

In the present study the influence of long photoperiod (LP, 16L:8D) and short photoperiod (SP, 8L:16D) on hepatic energy metabolism in the olive flounder (*Paralichthys olivaceus*) was investigated. Flounders were maintained under LP or SP conditions for 2 weeks then plasmatic and hepatic parameters were assessed. At the plasmatic level, the concentration of cortisol was enhanced in flounder maintained under LP compared to SP. Alkaline phosphatase (ALP), alanine aminotransferase (ALT) and aspartate aminotransferase (AST) enzyme activities in plasma also increased in LP flounder. There was no significant difference in plasma glucose levels between the two experimental groups. Plasma osmotic pressure, Na and Cl levels were significantly higher in LP compared to the SP group. In liver, a significant decrease of triglycerides together with an increase in glycogen was observed in the LP group. Hepatic *hsl* and *pepck* and muscle *hsl* mRNA expression in LP was significantly higher in the SP group. Overall the results indicate that the LP treatment caused a mild stress response and increased hepatic energy metabolism in the flounder, which in turn could affect osmoregulation. In conclusion, it would appear that LP treatment can adversely influence hepatic energy metabolism in adult olive flounder under fasting condition.

## Background

The influence of photoperiod on fish has been studied in respect to its effects on growth, reproduction and migration (Boeuf et al. 1999). Photoperiod has been considered as one of the most important growth promoting factors in several fish species, it affects not only feeding behavior but also physiological condition. The use of different light regimes is common in commercial aquaculture (Barimani et al. 2013; Sonmez et al. 2009; Taylor et al. 2005). A large number of literature has been reported a relationship between photoperiod and growth of fish, but the findings are often species specific. In some species, including barramundi *Lates calcarifer* (Barlow et al. 1995) and gilthead sea bream *Sparus aurata* (Tandler and Helps 1985), extending day length increases larval growth and survival; however, long photoperiod regimes result in physical stress and adverse growth effects in rainbow trout (*Oncorhynchus mykiss*) (Leonardi and Klempau 2003).

Olive flounder (*Paralichthys olivaceus*) is an economically important fish distributed in coastal China, Japan and South Korea (Dou et al. 2003; Xu et al. 2012). The increasing demand for flounde makes it an important candidate for commercial aquaculture. Different light regimes have been used to regulate the growth and survival of larval flounder (Dou et al. 2003; Moustakas et al. 2004), but the effect of photoperiod on metabolism in adult flounder is unstudied.

The flounder can adjust its physiology and metabolism to modifications in photoperiod and food availability, and these adjustments may occur in anticipation of the change, allowing flounders to keep homeostatic responses needed for survival in different conditions (Lu et al. 2007). The liver is an essential metabolic organ for growth in fish, it stores lipid and glycogen when energy and food are in sufficient supply and degrades and releases them into the blood during fasting (Barcellos et al. 2010). Phosphoenolpyruvate carboxykinase (*pepck)* and hormone-sensitive lipase (*hsl*) catalyze glycogen and lipid metabolism, respectively and contribute to maintain energy homeostasis during food deprivation. In the wild flounder face periods of starvation during overwintering, spawning and migration. In aquaculture practice, flounders are also exposed to short-term food deprivation during sorting, transportation and when they are temporarily maintained in holding tanks. What is more, artificial photoperiod manipulation may also alter physiologic metabolism and affect survival of flounders.

Therefore, to understand the possible involvement of photoperiod on metabolic effects in flounder, we conducted an experiment by exposing adult olive flounder to a long (16L:8D, LP) and short (8L:16D, SP) photoperiod. First we compared the concentration of cortisol, alkaline phosphatase (ALP), alanine aminotransferase (ALT) and aspartate aminotransferase (AST) in the plasma. Then we analyzed the glucose, glycogen and triglyceride levels in plasma and liver tissue and finally we investigated transcripts associated with lipolysis and glycogen metabolism, hepatic *pepck* and *hsl* mRNA expression, respectively. The aim of this study was to investigate the influence of long photoperiod manipulation on energy metabolism and the general physiology of olive flounder.

## Methods

### Ethics statement

All animal procedures were approved by the Animal Ethics Committee of Shanghai Ocean University (Shanghai, China) and complied with the Guidelines on Ethical Treatment of Experimental Animals set by the Ministry of Science and Technology, China.

### Animal

Gynogenetic olive flounder were produced as previously described (Liu et al. 2012) and were reared until they were approximately one-year-old at the Central Experimental Station of Chinese Academy of Fisheries Sciences (Beidaihe, Hebei, China). The experiment was conducted at the same location on September 15–30, 2012. A total of 18 gynogenetic olive flounder (body weights: 600 ± 50 g) were randomly distributed between 18 tanks in a flow-through, filtered seawater circuit at 20 ± 1 °C. Black plastic light-proof curtains surrounded each set of tanks and artificial illumination was provided with white fluorescent lamps. Mean light intensity was approximate 40 lux near the bottom in the centre of the seawater tanks. Fish were starved throughout the experiment.

### Animal experiments

Fish were acclimated under short photoperiod (8L:16D, SP; n = 6), middle photoperiod (12L:12D, MP; n = 6) and long photoperiod (16L:8D, LP; n = 6) for 2 weeks and then removed from each photoperiod tank without anaesthetic. All fish were stunned by percussion and humanely killed. Blood was collected within 90 s of death through the caudal blood vessels. Blood samples were aliquoted into ammonium-heparinized tubes and plasma was obtained by centrifugation for 5 min at 13,000*g*. Liver and muscle tissues were removed and snap frozen in liquid nitrogen for subsequent RNA extraction or biochemical measurement.

### Cortisol measurement

The level of cortisol in plasma was measured using a commercial RIA kit purchased from Beijing North Institute of Biotechnology (Beijing, China). The detection limit for cortisol was 2 ng/mL and the intra- and inter-assay coefficients of variation were 10 and 15 %, respectively. Samples were analyzed in triplicate within one assay to avoid inter-assay variations.

### Analysis of plasma osmolality and ions concentration

Osmolality was measured using a vapor pressure micro-osmometer (Vapro 5520 Wescor, Logan, USA), sodium concentrations were determined by atomic absorption spectrophotometry (Thermo Elemental Solaar S4, Winsford Cheshire, UK), chloride concentrations were analyzed by electrode titration (Corning Chloride Analyzer 925, Corning, NY).

### Measurement of plasma and liver metabolites

Glucose, glycogen and triglyceride levels in plasma and liver of fish were measured using commercial kits from Nanjing Jiancheng Bioengineering Institute (Nanjing, China) according to the manufacturer’s manuals. Methods were adapted with 96-well plates and values were determined spectrophotometrically using a microplate reader (Synergy, BioTek, Winooski, VT). Enzyme activities of ALP (AMP buffer method), ALT (IFCC method) and AST (IFCC method) in plasma were measured on an automated BS200 chemistry analyser (Mindray, Shenzhen, China).

### Gene expression analysis by real-time PCR

Total RNA was extracted from tissues using Trizol reagent (Invitrogen, Carlsbad, CA, USA) and treated with RQ1-DNAse (Promega, Madison, WI, USA) to remove DNA contamination. The concentration of RNA was measured using a NanoDrop ND-2000 Spectrophotometer (Wilmington, DE, USA). RNA integrity was confirmed by denaturing agarose gel electrophoresis. Total RNA (1 μg) was reverse transcribed into cDNA using Superscript II reverse transcriptase (Takara). Gene expression levels were determined by real-time quantitative RT-PCR using an ABI 7500 Real-Time PCR system (Applied Biosystems, Foster City, CA). Relative quantification of the target gene transcripts was done using *β*-*actin* gene expression as the reference gene. Specific primers for *hsl*, *pepck* and β-actin transcripts were designed using flounder mRNA sequences for *hsl* (AB828672), *β*-*actin (*AF135499) and *pepck* using Primer Premier 5.0 software. The sequence of the olive founder *pepck* gene was obtained from GEO database GSE47556 (Kondo et al. 2014). Primers were commercially synthesized (Sangon Biotech, Shanghai, China) (Table 1), and validation of primers for qRT-PCR were performed using standard ABI protocols. Thermal cycling was initiated by incubation at 95 °C for 5 min using hot-start Taq DNA polymerase; 40 PCR cycles were performed, and consisted of heating at 95 °C for 15 s, followed by 60 °C for 30 s and 72 °C for 30 s. Following the final PCR cycle, melting curves were systematically monitored to ensure that only one fragment was amplified.

**Table 1:** Primer pair sequences used for real-time PCR amplifications

RT-PCR primer	5′–3′	Annealing temperature (°C)
HSL F	ACAGCAGTCACGGTTTGGT	60
HSL R	AGAGGAAGGCGTAGAGGGA	
PEPCK F	GAACGGCTTCTTCGGTGT	60
PEPCK R	AAGCGGGAGTTGGGGTG	
β-actin F	GGAAATCGTGCGTGACATTAAG	60
β-actin R	CCTCTGGACAACGGAACCTCT	

*HSL* hormone-sensitive lipase; *PEPCK* phosphoenolpyruvate carboxykinase

### Statistical analysis

The 2^–ΔΔ^ Ct method was used to analyze the real-time PCR data (Livak and Schmittgen 2001), and amplified transcripts were expressed as the fold change relative to the mean value of the SP group. All data were presented as the mean ± SEM and statistical differences were detected using an unpaired Student’s t test using SPSS software version 18.0 (SPSS Inc., Chicago, IL). The cut-off for significance was p < 0.05.

## Results

### Mass and the concentration of cortisol in flounder

No mortality or disease was observed in either group of fish during the experiment. The mass of flounders at the end of the experiment was significantly lower (p < 0.05) than the initial body weight, and no significant difference in mass occurred between two groups (data not shown).

**Fig. 1:**
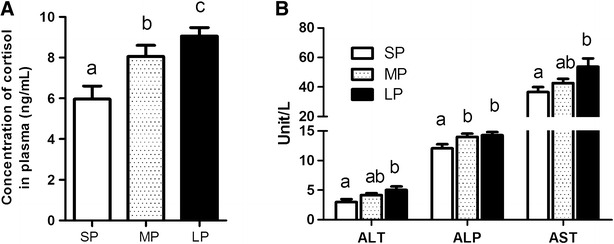
The concentration of cortisol (**A**) and three different biochemical parameters (**B**) alkaline phosphatase (ALP), alanine aminotransferase (ALT) and aspartate aminotransferase (AST) in plasma of flounders. Data are presented as mean ± SEM, N = 6. *Different letters* above *bars* mean significant differences among the three groups

Plasma cortisol levels in flounder acclimated to SP, MP and LP are shown in Fig. 1A. Photoperiod significantly affected stress indicators in flounder and cortisol was significantly increased in LP (9.07 ± 0.41 ng/mL) compared with SP (5.96 ± 0.64 ng/mL) and MP (8.06 ± 0.55 ng/mL) flounder (P < 0.01).

### ALP, ALT and ASP activity in plasma of flounders

Three serum biochemical parameters, ALP, ALT and ASP, that are associated with liver damage were analyzed (Fig. 1B). LP treatment significantly increased the activity of ALP (2.98 ± 0.48 vs. 5.02 ± 0.58 unit/L, P < 0.05), AST (12.08 ± 0.69 vs. 12.48 ± 0.55 unit/L, P < 0.01) and ALT (36.56 ± 3.34 vs. 53.76 ± 5.68 unit/L, P < 0.05) relative to the SP group. The data of ALT (4.18 ± 0.29 unit/L) and AST (42.73 ± 2.86 unit/L) in MP showed no difference compare to SP and LP, while ALP (14.02 ± 0.56 unit/L) showed significant increase than SP group.

### Osmolality and ion concentration in plasma of flounders

**Fig. 2:**
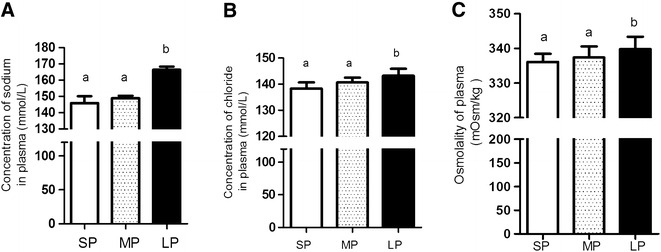
Ion parameters in plasma in two groups of flounders. The concentration of sodium (**A**), chloride (**B**) and osmolality of plasma (**C**). Data are presented as mean ± SEM, N = 6. *Different letters* above *bars* mean significant differences among the three groups

The concentration of Na^+^ and Cl^−^ in plasma is shown in Fig. 2. LP caused a significant increase in plasma Na^+^ and Cl^−^ (P < 0.05) relative to the SP and MP group (Fig. 2A, B). Elevated level of plasma osmolality (P < 0.05) was observed in LP compared with SP and MP flounder (Fig. 2C).

### Glucose, glycogen and triglycride in blood and liver tissue

**Fig. 3:**
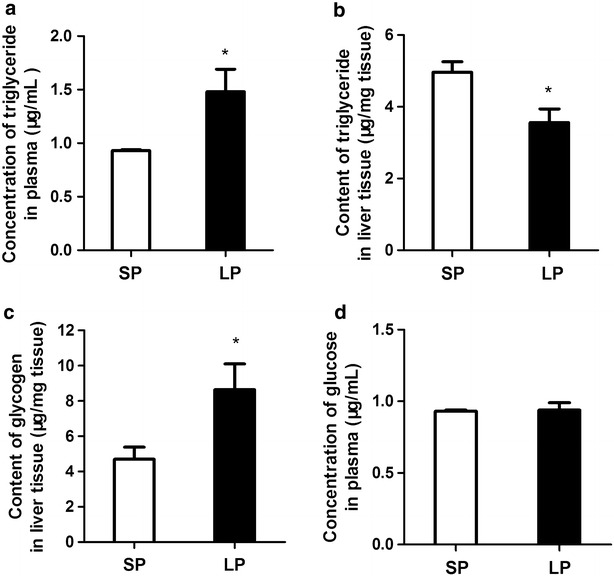
The blood and liver metabolic parameters in two groups of flounders. Content of triglyceride in plasma (**a**) and liver tissue (**b**), content of glycogen in liver tissue (**c**), concentration of glucose in blood (**d**). Data are presented as mean ± SEM, N = 6. *Asterisk* P < 0.05 indicates significant difference between the two groups

From the results of detected above, significant differences were mainly observed in LP and SP group, and the data of MP were in intermediate range between SP and LP group, so next analysis was focused on LP and SP group. Plasma and liver metabolites in flounder acclimated to modified photoperiod are shown in Fig. 3. The concentration of triglyceride in plasma was significantly higher (p < 0.05) in the LP group relative to the SP group (Fig. 3a). In contrast, triglycerides in the liver of the LP group was significantly lower (p < 0.05) than the SP group (Fig. 3b) and glycogen in the liver showed a reverse trend (Fig. 3c). There was no significant difference in plasma glucose between SP and LP groups (Fig. 3d).

### mRNA expression of *hsl* and *pepck* in the liver and muscle of flounder

**Fig. 4:**
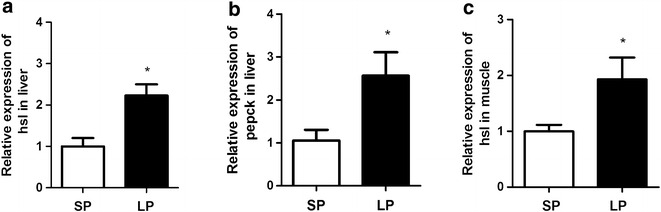
Relative expression level of hormone-sensitive lipase *hsl* (**a**) and Phosphoenolpyruvate carboxykinase *pepck* (**b**) gene expression in liver, and hsl expression in muscle (**c**). Relative expression data was calculated by the method 2− ΔΔCt, Data are presented as mean ± SEM, N = 6. *Asterisk* P < 0.05 indicates significant difference between the two groups

Expressions of *hsl* and *pepck* mRNA in the liver are presented in Fig. 4. Both *hsl* (Fig. 4a) and *pepck* mRNA (Fig. 4b) expression in the liver of the LP group was significantly higher than those in SP group (P < 0.05). Expression of *hsl* mRNA in the muscle of the LP group was also significantly higher (P < 0.05) than that in SP group (Fig. 4c).

## Discussion

The artificial extended photoperiod treatment for flounders presents physiological challenges with respect to osmoregulation and hepatic metabolism when food are not available in this study. These physiological challenges are revealed by the stress response detected in flounder exposed to a LP. In this study we observed that LP manipulation resulted in a mild stress response in flounder. The level of plasma cortisol was 9.07 ± 0.41 ng/mL in LP, which is lower than the 25 ± 4.4 ng/mL previously reported in flounders exposed to transportation stress (Hur et al. 2007). The lower levels of cortisol detected in the present study suggests that LP is a milder stressor than transportation. The significant increase in plasma cortisol in flounder exposed to LP agrees with the results of a study with African sharptooth catfish *Clarias gariepinus*, exposed to a long photoperiod (6D:18L) relative to those reared under shorter periods of light (24D:00L and 18D:06L) (Almazán-Rueda et al. 2005).

AST and ALT are aminotransferases present in the liver and spleen. Their plasma levels are low when animals are healthy, but increase when animals are stressed or when tissue necrosis occurs (Wells et al. 1986; Jung et al. 2003). ALT and AST leak into the circulation when liver cells are damaged and high levels of these enzymes in the blood are an indicator of liver damage. In this study we observed increased level of enzymes (AST, ALT, ALP), suggesting LP may exert adverse effect on liver metabolism in flounder. The results of the present study are similar to previous studies in which flounder were stressed by food deprivation or metal pollution (Park et al. 2012; Oner et al. 2008).

For seawater acclimation fish, gills absorb water and excrete sodium and chloride to maintain osmotic balance of blood; a highly energy-consuming process (Bœuf and Payan 2001; Iwama et al. 1997). Plasma osmolality is a useful marker to determine the effectiveness of osmoregulatory regulation. One of the main energy costs of osmoregulation is ion pumping (Urbina and Glover. 2015). Therefore, the energy state will affect the function of the gill and the concentration of ions in fish plasma. In the current study the higher Na, Cl level and osmolality in plasma of the LP group maybe revealed low energy availability resulting both from food deprivation and photoperiod manipulation. The results of the present study were consistent with the significantly higher levels of plasma Na and Cl in tilapia (*Oreochromis niloticus*) that were food-deprived (Vijayan et al. 1996). Increased plasma Na and Cl were also reported in striped jack (*Pseudocaranx dentex)* exposed to a long photoperiod (16D, 8L) when compared to a short photoperiod (8L, 16D) (Pavlidis et al. 1999).

Liver is the main organ involved in glycogen/glucose turnover in fish, and during fasting its metabolism is enhanced so that glucose can be made available for other metabolic and osmoregulatory process (Blasco et al. 2001; Klee et al. 1990). In the present study the level of glucose in blood showed no difference between the LP and SP groups and a similar observation was made in the silver catfish (*Rhamdia quelen)* fasted for 14 days d (Barcellos et al. 2010). Glyconeogenesis process means to produce glucose from non-carbohydrate substrates (Klover and Mooney 2004). *Pepck* catalyzes the rate-limiting step in hepatic glyconeogenesis. Glycogen metabolism in liver tissue is the principal energy source in both vertebrates and invertebrates, especially during environmental fluctuation such as food deprivation (Kullgren et al. 2010; Mariano et al. 2009). In this study, the glycogen content of the liver was significantly higher in the LP group relative to the SP group, implying that the former group have a higher glyconeogenesis capacity. Increased cortisol concentration combined with higher level of hepatic *pepck* gene expression and glyconeogenesis is consistent with the result of Vijayan et al. (1994), who reported that cortisol treatment increases glyconeogenesis in rainbow trout (*Oncorhynchus mykiss*) hepatocytes. The results of the present study support the notion that cortisol-induced glyconeogenesis is important for the maintenance of glucose levels during energy demand in flounders (Vijayan et al. 2003).

Lipolysis of triglyceride stored in the liver results in the liberation of glycerol and nonesterified fatty acids that are released into the blood for use by other organs (Reshef et al. 2003). During time of energy deprivation in fasting, the LP group of flounders have higher e energy requirement than SP group, and when food is unavailable this need is met by increased hepatic lipolysis. The triglyceride content of the liver in the LP group was significantly lower than the SP group, while the content of triglycerides in blood showed reversed tendency. H*sl*, the rate-limiting enzyme for lipolysis, participates in the hydrolysis of triacylglycerol to generate fatty acids and glycerol for use as an energy substrate by other organs (Mulder et al. 1999). The amount of triglyceride in liver of the LP group was significantly lower than that of SP group, and this with the higher level of *hsl* mRNA expression in liver and muscle supports the idea that LP flounder have enhanced lipid degradation to meet their increased energy demand. This result is in line with the study of Tian (2013), who reported increased *hsl* mRNA levels in the liver of Nile tilapia (*Oreochromis niloticus*) during fasting.

Studies in mammal with liver-specific *pepck* knock-out mice found a dramatic increase in hepatic triglycerides in the liver of *pepck* null mice (Burgess et al. 2004), suggesting that glycogenesis involving PEPCK is linked with lipolysis in liver tissue. In our study we observed significantly higher *pepck* mRNA and enhanced glycogen associated with decreased triglyceride in liver of LP flounder, which suggests that utilization of triglyceride reserves in the liver was significantly higher in sthe LP flounders relative to the SP flounder.

In conclusion, our results suggest that long photoperiod manipulation result in mild stress response and increased energy requirement in flounder under conditions of food deprivation, and the shortage of energy affect osmoregulatory function. LP increased lipolysis of triglyceride and glycogenesis in the liver in response to the increased energy requirements. One of the main features of aquaculture is feeding at the maximum possible rate, so our results may not have relevance for normal feeding practices in flounder aquacultures. Nonetheless, since aquaculture practice inevitably involve periods of food deprivation, such as sorting, transportation and holding in temporary tanks, if they are simultaneously exposed to LP it may have a negative effect and should be taken into account during aquaculture of flounder.
